# Modelling amorphous computations with transcription networks

**DOI:** 10.1098/rsif.2009.0014.focus

**Published:** 2009-05-27

**Authors:** Zack Booth Simpson, Timothy L. Tsai, Nam Nguyen, Xi Chen, Andrew D. Ellington

**Affiliations:** 1 Institute of Cellular and Molecular Biology, University of Texas at Austin, Austin, TX, USA; 2 Center for Systems and Synthetic Biology, University of Texas at Austin, Austin, TX, USA; 3 Department of Computer Science, University of Texas at Austin, Austin, TX, USA

**Keywords:** transcription, logic gate, oscillator, amorphous computation, switch, complementary metal oxide semiconductor

## Abstract

The power of electronic computation is due in part to the development of modular gate structures that can be coupled to carry out sophisticated logical operations and whose performance can be readily modelled. However, the equivalences between electronic and biochemical operations are far from obvious. In order to help cross between these disciplines, we develop an analogy between complementary metal oxide semiconductor and transcriptional logic gates. We surmise that these transcriptional logic gates might prove to be useful in amorphous computations and model the abilities of immobilized gates to form patterns. Finally, to begin to implement these computations, we design unique hairpin transcriptional gates and then characterize these gates in a binary latch similar to that already demonstrated by Kim *et al*. (Kim, White & Winfree 2006 *Mol. Syst. Biol.* **2**, 68 (doi:10.1038/msb4100099)). The hairpin transcriptional gates are uniquely suited to the design of a complementary NAND gate that can serve as an underlying basis of molecular computing that can output matter rather than electronic information.

## 1. Introduction

Chemists and biologists are in the process of creating many de novo implementations of chemical computation and control logic. Given the rich history of such development by electrical engineers in the nineteenth and twentieth centuries, it is worthwhile to consider key aspects of this history and translate this into the domain of molecular computing. The evolution of electronic amplifiers and gates is of particular interest. For example, the workhorse of electrical gate design, the complementary metal oxide semiconductor (CMOS), embodies several important lessons regarding power management and signal-to-noise optimization that should translate to molecular computers.

One of the most likely avenues for developing modular and scalable molecular computers is by engineering DNA to act as a logical nanodevice. For example, Winfree and co-workers have engineered DNA machines that perform logical operations using sequence hybridization as an input and sequence release or production as an output (Seelig *et al*. 2006*a*). The Winfree laboratory has constructed autocatalytic cascades in which small amounts of input DNA signal are amplified as the machine devolves to a lower energy (higher entropy) state (Seelig *et al*. 2006*b*; Zhang *et al*. 2007). The function of the autocatalytic cycle can be further modulated by allosteric DNA molecules that adopt either catalytically active or inactive conformations based on additional sequence inputs (Zhang & Winfree 2008). Similar machines can be constructed, in which the generation of sequence outputs is not owing to the release of hybridized oligonucleotides, but rather to the activation of transcriptional units (Kim *et al*. 2006). Using transcriptional logic gates, a bi-stable circuit based on cross-interfering transcriptional logic gates has been demonstrated. A great advantage of all of these logical DNA machines is that the interactions and energetics of the machines can be finely programmed based on the known energetics of nucleic acid secondary structure formation (Dirks *et al*. 2004).

We reasoned that immobilization of transcriptional gates might lead to similar logical calculations on surfaces, but with the outcome being a large-scale computation: pattern formation. In order to test this hypothesis, we have developed a diffusion-based model for transcriptional networks and have shown that a variety of patterns can arise from even simple assemblies of a limited number of transcription gates. Sensitivity analyses performed on the amorphous computations (Abelson *et al*. 2000) performed by the networks reveal that even minor changes in variables relating to the spatial and temporal coupling of the gates can have remarkably robust impacts on the resultant patterns. Some of the simple DNA gates required to execute such surface-based, amorphous computations have been experimentally tested.

## 2. Conceptualization

### 2.1. Equivalences between electronics and chemistry

**Figure 1.**
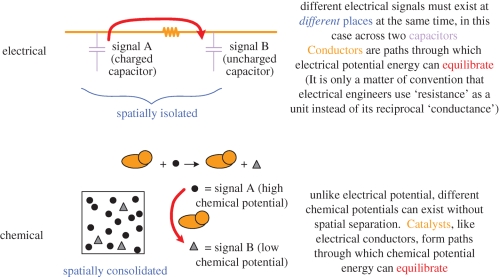
Chemical equivalences to electrical flow. In this and subsequent diagrams, standard electronic symbols are used. In the ‘chemical’ portion of the diagram, circles and triangles represent substrates and products. The orange oval represents an enzyme that lowers the activation energy for interconversion of these substrates and products. In electronics, signals are encoded by storing potential energy across a capacitor and conductors (wires and resistors) permit signals to equilibrate. By analogy, enzyme catalysts act in the same role as electrical conductors, permitting signals stored in chemical potential to equilibrate.

One of the main thrusts of ‘synthetic biology’ is to port existing engineering discipline into the practice of biotechnology. Because much computational and control theory has been developed by electrical engineers, it is useful to detail the analogy between chemical and electrical systems (figure 1).

Electrical engineers encode signals by varying the concentration of charge per unit capacitance. Electrical circuits are networks of conductors (wires, resistors and other devices) that permit the equilibration of the signal carriers (electrons) between spatially separated capacitors in some predetermined, interesting manner. In contrast, chemical circuits encode signals by varying the concentration of non-identical reagents per unit volume. Chemical reagents can coexist in the same volume of space separated by an energy barrier that is unique to the reagents. Thus, in the same way that electrical circuits are built by bridging the energy barriers between capacitors with conductor networks, chemical circuits can be built by bridging the energy barriers between reagents with catalytic networks. However, the difficulty in scaling such chemical networks is that the energy barrier between any two reagents is typically unique to that pair of molecules and, therefore, a catalyst that bridges them must be similarly unique. While an electrical engineer can trivially build a scalable array of identical capacitors and wire them in some specific way, a chemist does not have an equivalent luxury. It is for this reason that much interest has been expressed in the use of nucleic acids as addressable chemical signals (Stojanovic & Stefanovic 2003; Kim *et al*. 2006), leading to a possible scenario of scalable chemical computing.

**Figure 2.**
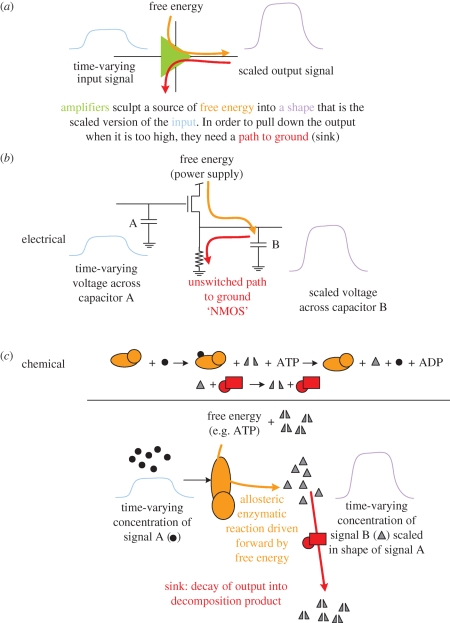
Amplifiers. (*a*) A technology-independent schematic of an amplifier (triangle). For simplicity, schematic amplifier diagrams usually show only the input and output signals (left and right of the triangle) and omit the connections for power supply (free energy) and ground (sink). (*b*) An example electrical implementation (NMOS) of an amplifier. An N-doped transistor is switched by the input A routing the free energy (power supply) to charge the output capacitor encoding signal B. An unswitched resistor constantly drains B enabling it to come low when the input falls. (*c*) An enzymatic amplifier. Input signals (black circles, different from the use of this symbol as a substrate as shown in figure 1) can allosterically control an enzyme (orange oval). The enzyme uses a source of free energy (ATP) to convert a lower energy substrate (half grey triangles) into a higher energy product that is also an active signal (full grey triangles). Another enzyme (red square) acts as the sink by accelerating the constant drain of the product back to its more chemically stable form. Note that the input chemical signal is *not* converted to the output chemical (the amplifier enzyme does not convert input signal into output product) but rather the *state* of input drives a reaction resulting in a similarly shaped state of the output.

Amplifiers (figure 2) are the base technology for signal processing, and it is therefore important to extend the chemical analogy to these devices. Amplifiers sculpt a source of free energy into the time-varying shape of the input signal. They do not pass along the input energy. Instead, amplifiers make a new output signal independent of the input but also proportional to it. Without the amplifier's ability to increase the gain of the signal using the source of free energy, only limited serial operations would be possible because the signal energy would be progressively consumed by each serial stage until it was depleted.

Equally important to the source of free energy is a sink (figure 2, red arrow). If it were not for a way to remove energy from the output, the output would be able to rise but never fall; an amplifier without a sink would be a one-shot-ever device.

The chemical analogue of an electrical amplifier (figure 2*c*) is an allosterically regulated catalyst that sculpts a source of free energy into the shape of a time-varying input signal. For example, the input signal might be encoded in the concentration of a molecule that allosterically regulated a kinase enzyme. The kinase would use free energy from ATP to phosphorylate an output reagent. A phosphatase could dephosphorylate the output and thereby act as the required sink. Such a kinase/phosphatase pair would ideally keep the concentration of phosphorylated output reagent in proportion to the concentration of the input reagent.

**Figure 3.**
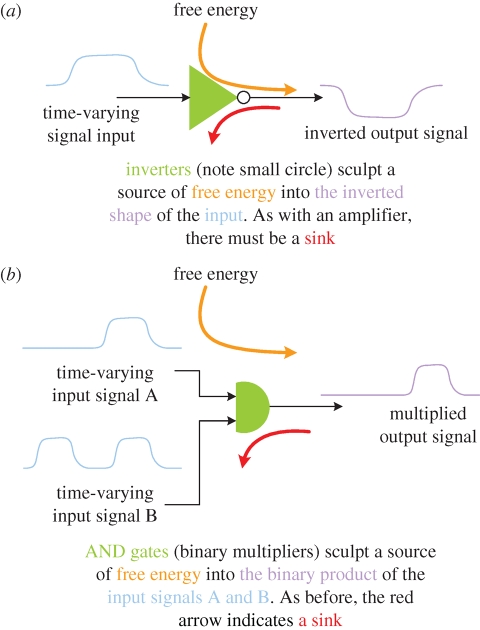
Gates. Gates are amplifiers that also perform some function transform of the input signals. (*a*) An inverter (note small circle on output of green triangle) is an amplifier connected so that the input shape is inverted (scaled by −1). (*b*) An AND gate binary multiplies two inputs resulting in an output only when both inputs are high.

Gates (figure 3) are not only amplifiers but also transform and reshape output signals in some way. For example, an inverter or a ‘NOT gate’ (figure 3*a*) multiplies the input signal by a negative number; an ‘AND gate’ (figure 3*b*) generates an output that is proportional to the multiplicative input of the two inputs. In chemical terms, an allosteric kinase enzyme that was coordinately regulated by two inputs could similarly be an AND gate. It is extremely important to appreciate that these gates, whether electronic or chemical, are amplifiers; without gain, only limited serial computational operations would be possible. The problem with the chemical gate as described is that its gain cannot subsequently be pulled down to zero without additional engineering.

**Figure 4.**
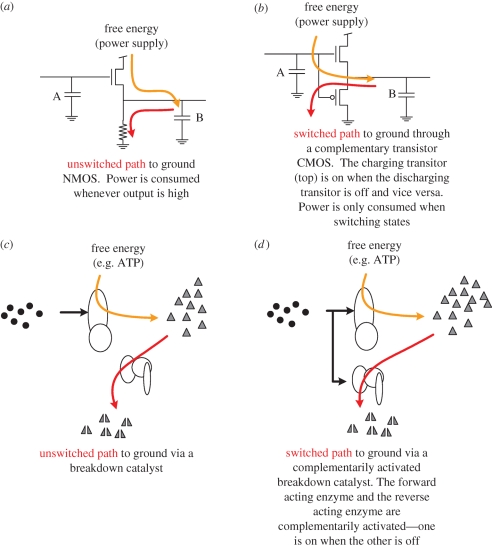
NMOS versus CMOS. (*a*) An electrical NMOS amplifier using a resistor that drains the output constantly is inefficient because when the input is high, the pull-up transistor is fighting the resistor, thus burning power straight to ground. (*b*) A complementary configuration where the drain path is now switched by a ‘p-doped’ semiconductor. This configuration is preferable because the charge and drain paths are always switched opposite to each other and therefore never compete. (*c*) The chemical analogue of NMOS uses an unswitched enzyme (red path) to constantly discharge the output. (*d*) A chemical analogue to CMOS where the drain enzyme is now allosterically controlled with opposite logic like its electrical analogue.

As discussed, amplifiers and gates require a sink path to pull the output low. The design of this sink is subtle. Early semiconductor computers implemented with MOS used the previously demonstrated method of tying all outputs to ground via resistive paths (figure 4*a*). In such a configuration, the charging circuit must be able to overpower the constant drain to ground whenever the output signal is driven high. This technology, called ‘NMOS’ (the ‘N’ stands for ‘negative doped’), has several disadvantages. First, whenever the output of the gate is high, it wastefully consumes power because the sink futilely shuttles charge to ground. Second, the time to charge and discharge the gate is not the same—the transition from high to low is quick while the transition from low to high is slower owing to the competing sink path. One can attempt to mitigate this discrepancy by increasing the conductance of the sink path, but only at the cost of more wasteful power dissipation in the high state. Third, the high and low states do not have the same signal-to-noise ratio (SNR). The high state is encoded with a large energy relative to the background noise, while the low state is encoded with low energy relative to the same background noise. As a result, the SNR of the high state is much greater than the SNR of the low state. In essence, ones and zeros are not created equally, and various engineering challenges arise from this complication—what electrical engineers sometimes call ‘asymmetric voltage transfer characteristics (VTCs)’ (Sedra & Smith 2004).

To avoid the disadvantages of NMOS, the computer industry converted in the late 1980s to what is called ‘CMOS’. CMOS switches the sink to ground in complement to the charging circuit (figure 4*b*)—when the charging circuit is conducting, the discharging circuit is disabled and vice versa. At no time is the power supply connected directly to ground and thus energy is only consumed in the transition from one state to another. CMOS also permits the charge and discharge times to be easily matched. Furthermore, CMOS permits encoding the two states, high and low, as positive and negative charges, respectively. Therefore, the zero and one states can have symmetric noise characteristics, and the aforementioned problems are correspondingly reduced.

The chemical analogue to NMOS (figure 4*c*) uses a catalyst that constitutively reduces the concentration of the output reagents. For example, Winfree and co-workers implemented a sink in their transcriptional circuits using RNase H (Kim *et al*. 2006). However, to our knowledge, no one has yet proposed an exact chemical analogue of CMOS.

### 2.2. Chemical amorphous computing

The term ‘amorphous computing’ (Abelson *et al*. 2000) has been coined to describe computations performed by spatially distributed masses of processors with the following features: all processors are identical, they may have limited computational abilities, they have no shared data bus, no shared clock, can communicate only locally and have no *a priori* knowledge of their spatial position within the collection. Informatically, multi-cellular organisms and colonies fit this amorphous computing description very well—clonal cells are identical processors, the internal transcriptional and translational state of cells can perform computation and the diffusion of intercellular molecules can implement local communication. However, cells are much more than just computers; they also fabricate within the same medium and, in consequence, demonstrate pattern-forming talents that are the envy of engineers.

**Figure 5.**
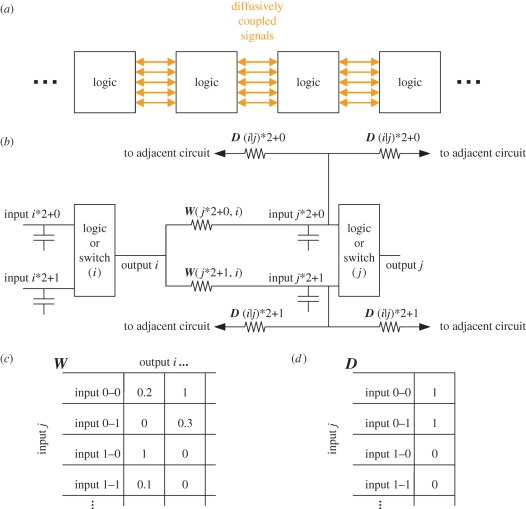
(*a*) Generalized scheme for one-dimensional diffusive amorphous computing. A series of identical logic gates is arrayed through space with the internal signals of each allowed to diffuse to adjacent areas. (*b*) An electrical schematic showing a subset of the logic in one of the amorphous logic units illustrating the interconnection between the output of one gate (*i*) and the two inputs of another gate (*j*). Each input (*j*) has a unit capacitor tied via a unique conductor path to the output of every other gate (*i*). The strengths of these interconnection paths are stored in (*c*) the matrix ***W***. Every signal is coupled to its adjacent spatial positions left and right via conductors which simulate diffusion. The diffusion constant for each node is stored in (*d*) the vector ***D***.

We propose a generic model for exploring biologically inspired amorphous computing (figure 5). A logic device is modelled by a fully connected set of *n* two-input NAND gates. The output of every gate (*i*) is connected to the inputs of every other gate (*j*) by a conductor network described by the (2*n* by *n*) matrix ***W***. Identical copies of these logical devices are distributed uniformly through space (for illustration purposes, in one dimension) at high density. The internal nodes of these logic blocks are coupled to the nearest neighbours by conductors that permit diffusion. The diffusive coupling between any two nodes is considered to be only a function of the signal carrier and is therefore able to be modelled by a list of diffusion constants in the vector ***D*** and can be visualized as sets of identical resistors travelling between the spatially distributed nodes as seen in the electrical equivalence diagram in figure 5*b*.

**Figure 6.**
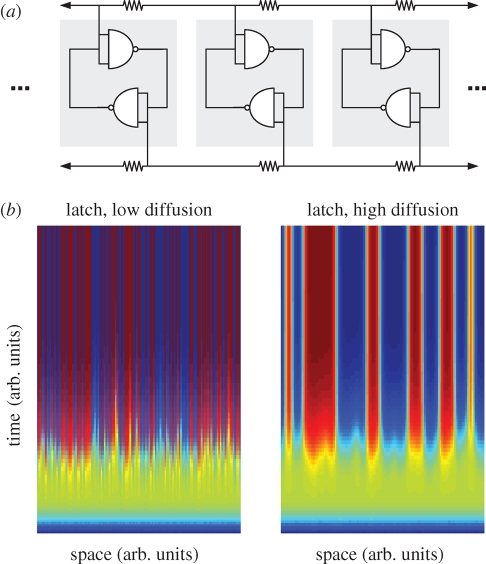
Spatial array of bi-stable latches. (*a*) Schema of a spatial array of bi-stable latches. Note the diffusion resistors top and bottom that couple each node to the adjacent positions. (*b*) Modelling the performance of immobilized, bi-stable latches. Simulations of the schema shown in (*a*) were carried out with noisy initial conditions. Colours (blue and red) represent the concentrations of each transcript; intermediate colours (green) indicate gradients between these transcripts. Each gate is arrayed in space along the *x*-axis (as in figure 5) in a circle (i.e. left connects to right). The performance of the array evolves through time as shown along the *y*-axis. Because the circuit is bi-stable, each gate commits to a fully blue or fully red final state based on the random initial conditions. Left—a simulation with no diffusion (***D*** = 0). Each volume of space commits randomly to blue or red with no regard for its neighbours. The feature sizes (the horizontal length of red or blue regions) are, therefore, determined by the binomial distribution. Right—same simulation with same initial conditions but with higher diffusion (***D*** = 1). Regions that by chance had more red or blue transcripts were able to recruit their neighbours to their state during the early evolution of the array. Ultimately, the gain of the latch forces each area to commit to blue or red as before, but recruitment leads the feature lengths to be significantly longer.

As an illustration of how amorphous gates can lead to simple pattern formation, consider an amorphous array of latches (figure 6*a*). A latch is a device in which two inverters are in feedback such that the output of each is trying to invert the other's input. Latches are bi-stable and will evolve into one of two ending states depending on the relative strength of the initial conditions on either side of the latch. If the initial conditions of the two sides are uninitialized, the latch acts as a coin toss—randomly committing to one or the other state. A one-dimensional amorphous collection of such uninitialized latches would create what amounts to a set of coin tosses laid side by side. In figure 6*b*, left, the blue and red colouring represents the latch committing to one state or the other. As time passes (bottom to top), it can be seen that each volume of space (one-dimensional pixel, *x*-axis wrapping on itself) commits to either a blue or a red state. A ‘feature’ in this space can be thought of as an area of common fate, for example, a long run of ‘reds’ makes a wide red feature. If the amorphous latches cannot communicate with each other, then the feature sizes will form a binomial distribution of coin-tosses. However, if the latches can communicate locally by diffusion, then the feature size increases. This effect can be thought of as a process of recruitment. As each volume of space begins to commit to either red or blue, it sends some of that signal to its neighbour. If its neighbour is still on the fence about which side to commit to, this influence may sway the decision. If, however, the neighbour is already in agreement, then the influence instead accelerates the commitment. By this recruitment process, areas that by chance happen to be enriched for one state, say red, will begin to exert a larger collective influence on their blue neighbours, thereby making even larger areas of agreement. The results can be seen in figure 6*b*, right. In dimensionless Monte Carlo experiments of this amorphous latch, it was determined that the mean feature size varies to the half power of the diffusion constant (figure S1, electronic supplementary material).

**Figure 7.**
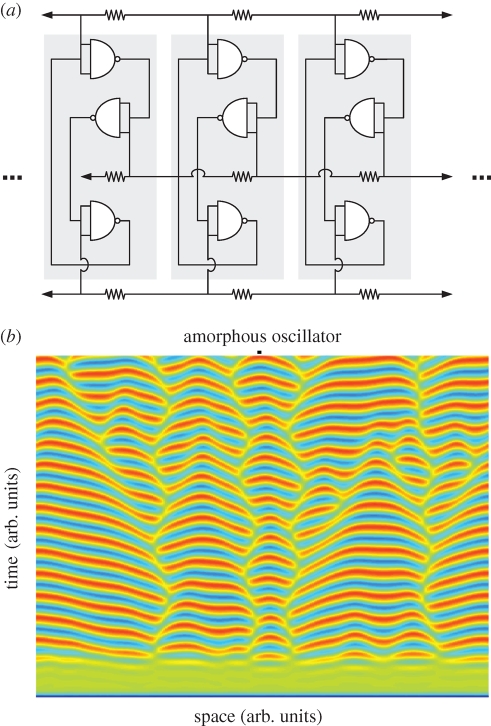
Spatial array of ring oscillators. (*a*) Schema of a spatial array of ring oscillators. This schema is identical to figure 6*a* but with an odd number of inverters making each circuit oscillate. Note the three stripes of conductors that couple the state of each node to its adjacent cells left and right. (*b*) Modelling the performance of an amorphous ring oscillator. Simulations were again carried out with noisy initial conditions. Colours are as in figure 6*b*. Because of the noisy initial conditions, each point in space begins in a random phase. Owing to diffusion, areas that by chance had similar phase begin to recruit their neighbours into the same phase. However, by chance it can occur that two areas end up with nearly 180° phase separation and the boundaries between these two regions begin to compete. Interestingly, oscillators near the phase boundaries run faster resulting in complex pattern formation.

With three inverters in series, an unstable logic network called a ‘ring oscillator’ is created (figure 7*a*). In the same way that an uninitialized latch commits to a random state, an uninitialized ring oscillator commits to a random phase. With no communication, each volume of space randomly chooses a phase, and no pattern is discernable. However, with diffusion, phase recruitment occurs in the same way that state recruitment does for latches. Such phase recruitment of coupled oscillators has been known since Huygens' experiments of 1665 and demonstrated by many others (a comprehensive review of which is given by Pikovsky *et al*. (2001), including a translation of Huygens' original letter describing the phenomena). Given enough time, an amorphous array of coupled oscillators will eventually fully phase synchronize; however, in the transition, they are capable of making patterns (figure 7*b*). The interesting discontinuities are caused by the fact that oscillators near a 180° phase boundary oscillate faster than those with no phase difference (see also figure S2, electronic supplementary material). While scalable chemical implementations for amorphous computing are still not available, we have used the kinetic model of Kim *et al*. (2006) to model a one-dimensional amorphous ring oscillator in order to determine the feature size. Simulations (figure S3, electronic supplementary material) reveal that the feature sizes are of the order of millimetres and synchronization times are of the order of tens of hours. These length and time scales suggest a variety of interesting applications for otherwise nanoscale computational devices whose ultimate output is a material (nucleic acid).

## 3. Implementation

### 3.1. Designing NAND gates for CMOS

**Figure 8.**
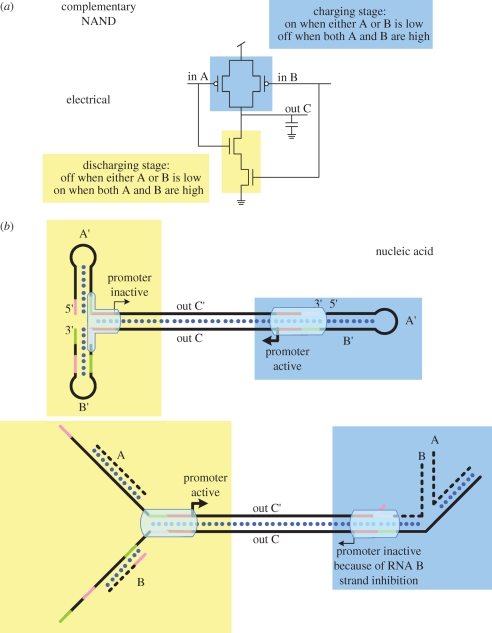
NAND gate. (*a*) An electrical implementation of a ‘not and (NAND)’ gate. The charge stage (blue) drives the output when either signal A or B is active. The discharge stage (yellow) pulls the output down in the complementary logic, specifically when A and B are high. (*b*) Proposed nucleic acid analogue of a ‘CMOS’. Signals are encoded as complementary nucleic acid strings where the signal is said to be high when an RNA transcript is in high concentration and low when the antisense complement of that string is in high concentration. The transcriptional gate is broken into two, independently controlled promoters (yellow and blue areas) on opposite sides of a DNA template. The charge stage (blue) completes the promoter when signal A or B is low absent (i.e. when A′ and B′ are high). Conversely, the discharge stage is on only when signals A and B are high. While this analogue is imperfect because transcription is not self-limiting, additional modifications of polymerases or transcript, as proposed in the text, would render it equivalent to CMOS.

To translate from the electronic schematic (figure 5) to a molecular computer, we propose to further optimize one-piece transcriptional gates as logic blocks (Kim *et al*. 2006). In particular, we have conceptualized the function of a transcriptional switch that will act as a complementary NAND gate (figure 8). In this gate, two promoters are present: one of which drives the production of output transcript C and one of which drives the production of its complement C′. Since C is complementary to C′, the production of one can cancel the action of each other. On the left-hand side of the diagram, regulatory elements function as an AND gate to activate the C′ output. Two hairpins sequester portions of the promoter, keeping it quiescent. Only in the presence of both A and B are the hairpins destabilized, allowing the promoter to be completed and the sequence C′ to be made. On the right-hand side, the regulatory elements function as the complement: NAND. A single hairpin sits adjacent to a fully functional promoter that is producing sequence C. Only in the presence of both A and B is this hairpin opened, allowing invasion of the promoter and its subsequent inactivation.

There are several interesting features of the proposed gate structure. First, using complementary signals C and C′ permits the possibility of achieving what electrical engineers would call a ‘symmetric static VTC’ (Sedra & Smith 2004), implying equal driving capabilities in both directions and thus making noise margins equal for both logic states. Second, the fact that the regulatory sequences are made of RNA while the gate itself is made of DNA is useful for the design of the right-hand side of the gate: invading RNA will both bind more tightly to DNA and of necessity inactivate the promoter. The difference in chemistry between regulatory elements and templates should also prove useful for draining signals from the system as the RNA can be differentially degraded with ribonucleases (similar to the use of RNase H by Winfree and co-workers (Kim *et al*. 2006)).

There is significant difference between the electrical CMOS design and the proposed DNA analogue as depicted in figure 8. Electrical CMOS gates switch the conduction between the power supply and the output capacitor, permitting them to equilibrate; that is, the output potential can never exceed the power supply. This is not the case with the proposed DNA analogue. In the DNA switch, when the promoter activates, the polymerase will produce either C or C′ until either the gate changes state or, in the extreme limit case, the power supply is exhausted. Therefore, the proposed design is not a perfect analogue.

There are several theoretical solutions that might improve the design and make it a better mimic of CMOS. The simplest solution is to use ribonucleases to drain the system (Kim *et al*. 2006). However, this solution would negate the power benefits of CMOS; indeed, it would double the power problems as now both high and low states would consume power through sink paths. A more preferable solution would be to engineer a way to have the output self-limit the gate. One possible implementation of a self-limiting gate would be to somehow have sequence-specific inhibition of each individual gate. For example, different polymerases and promoters could be used for each gate, and the RNA transcripts made from each promoter would contain an aptamer that could inhibit the polymerase specific to that gate. Aptamers have previously been selected against a number of polymerases, including DNA polymerases, reverse transcriptases and RNA polymerases (e.g. Biroccio *et al*. 2002). As a transcript built up, it would turn off the polymerase that led to its production. While this solution is reasonably straightforward, it reduces the hypothetical scalability to only a handful of carefully chosen polymerases (e.g. T7, T3 and SP6) and their cognate aptamers. A fully general-purpose complementary address-specific self-inhibiting transcriptional gate is, therefore, still elusive, but could potentially be modelled on the ingenious DNA : enzyme conjugates proposed by Gianneschi & Ghadiri (2007).

Beyond these concerns, the practical implementation of this gate using ribonucleases or custom polymerases will probably require a great deal of optimization to avoid potential problems, such as strand migration at the left-hand promoter that may lead to promoter completion and leaky transcription in the absence of A and B. The energetics of the gate will have to be carefully poised to ensure that both A and B are necessary to activate the right-hand promoter, and conversely that in the absence of A, the hairpin will fold and displace B, avoiding hysteresis in promoter function. Finally, the presence of B and B′ complementary sequences within the hairpins at each end of the gate could lead to intra- or intermolecular interactions that degrade the performance of the gate. Nonetheless, we believe that even the conceptualization of this gate structure moves forward attempts to design and model ‘biotronic’ circuitry.

### 3.2. Experimental implementation of one-piece transcriptional gates

**Figure 9.**
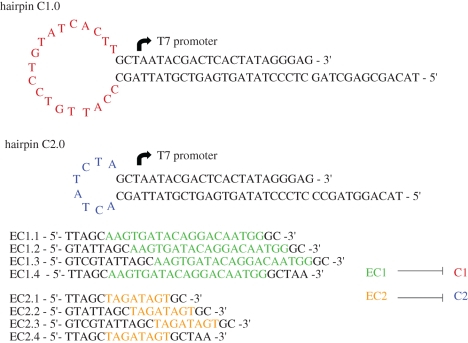
Constructs used to test one-piece transcriptional switches. Green regions of the oligonucleotide inhibitors are complementary to the red loop in C1.0. Orange regions of the oligonucleotide inhibitors are complementary to the blue loop in C2.0. Uncoloured sequences outside these core interaction regions can also potentially contribute to binding and inhibition of either C1.0 or C2.0.

In order to determine the feasibility of constructing a complementary NAND gate similar to the one described in figure 8, as a first step we attempted to generate hairpin promoter structures that could be regulated by sequence inputs (figure 9). *In vitro* transcriptional gates have previously been constructed (Kim *et al*. 2006), but these gates were made from several pieces of DNA, and promoter inhibition occurred when an antisense RNA bound to and dissociated a portion of the promoter. While this strategy was effective for homogeneous solution phase assays, it would be less useful for the immobilized circuits that we envision, where transcription potential has to be localized at particular points on a surface. We preserve the ability to act upon the nanomachine via a toehold, but our toehold is found within the loop sequence of the hairpin. It is likely that the kinetics of one-piece transcriptional gates is much quicker and more readily reversible than the kinetics of multi-piece gates. Similar hairpin structures have previously been used for sequence-specific control of translation (Isaacs *et al*. 2004) and for DNA nanomachines (Turberfield *et al*. 2003; Green *et al*. 2006).

**Figure 10.**
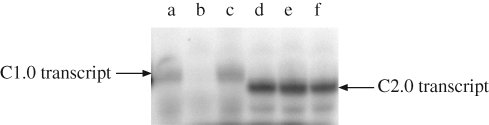
Inhibition of transcriptional switches. C1.0 (lanes a–c) and C2.0 (lanes d–f) were transcribed in the presence of either no inhibitor (lanes a and d), EC1.3 (lanes b and e) or EC2.3 (lanes c and f). EC1.3 is complementary to C1.0, and EC2.3 is complementary to C2.0. Sizes of products were determined relative to standards (data not shown).

Two different hairpin T7 RNA polymerase promoter structures were designed: one with a larger (C1.0, 19 nt) loop and another with a shorter (C2.0, 8 nt) loop. When transcribed *in vitro*, these structures produced RNA molecules of the predicted sizes, 18 nt for C1.0 and 16 nt for C2.0. These functional transcription units were then challenged with a variety of antisense DNA oligonucleotides complementary to the loop regions and portions of the stem structures. The DNA oligonucleotides were in essence surrogates for the RNA transcripts that would be involved in the regulation of the gates in circuits. Only one combination, C1.0 with EC1.3, was found to effectively inhibit transcription (figure 10, results with other inhibitors not shown). However, the inhibition proved to be extremely effective, with only 8.5 per cent read-through (relative to no inhibitor) observed after 1 h of transcription and only 2 per cent within the first 15 min. Given that the basic model for regulated, hairpin promoter structures has proven tractable, several of these can be ganged together to create gates similar to those shown in figure 8. In order to optimize the kinetic performance of these gates, it should be possible to vary the length of the internal (loop) toeholds (Dirks *et al*. 2004), the use of auxiliary nucleic acids to catalyse invasion (Bois *et al*. 2005) and potentially the buffers used for transcription.

**Figure 11.**
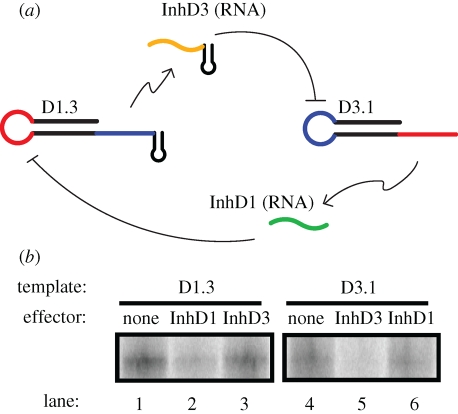
Mutual inhibition of two hairpin promoters. (*a*) Schematic of the two mutually inhibitory hairpin promoters and their products. RNA InhD3 (which is the product of hairpin promoter D1.3) is complementary to the loop of hairpin promoter D3.1 whose product, InhD1, is complementary to the loop of hairpin promoter D1.3. The small hairpins placed at the 3′ termini of D1.3 and InhD3 reduce aberrant transcription products. (*b*) The hairpin promoters are subject to cross-regulation. Left panel: lane 1 shows the radiolabelled transcription product of promoter D1.3 in the absence of effector, lane 2 is the same transcription reaction in the presence of InhD1 (10-fold excess) and lane 3 is in the presence of InhD3 (10-fold excess). Right panel: lanes 4–6 show a similar experiment with promoter D3.1. Lane 4 contains no effector, lane 5 contains a 10-fold excess of InhD3 and lane 6 contains a 10-fold excess of InhD1.

Based on these results, we designed two mutually inhibitory hairpin promoters and accompanying templates (termed D1.3 and D3.1, respectively) that could participate in a bi-stable latch (figure 11*a*). As shown in figure 11*b*, the purified transcription product of D3.1 (named InhD1) potently inhibited the efficacy of promoter D1.3 (lane 2) but not promoter D3.1 (lane 6), indicating that this inhibition is sequence-specific. Similarly, the product of D1.3 (named InhD3) potently inhibited D3.1 but not D1.3 (lanes 2 and 5). These results validate the initial design principles set out earlier and pave the way to the construction of more complex molecular computers.

### 3.3. Model for immobilization and pattern formation

In order to adapt transcriptional gates shown in figures 8 and 11 to the types of pattern formation modelled in figures 6 and 7, we envision that the gates might be surface immobilized (for example, via a pendant biotin moiety on a two-dimensional surface coated with streptavidin), similar to Frezza *et al*. (2007). The entire surface would be flooded with T7 RNA polymerase and nucleoside triphosphates in an appropriate buffer. The RNA transcripts produced from the transcriptional gates would diffuse between the different logic blocks, activating and/or inactivating them.
